# In Silico and In Vitro Anti-*Helicobacter Pylori* Effects of Combinations of Phytochemicals and Antibiotics

**DOI:** 10.3390/molecules24193608

**Published:** 2019-10-07

**Authors:** Pedro Fong, Chon-Hou Hao, Chi-Cheng Io, Pou-Io Sin, Li-Rong Meng

**Affiliations:** School of Health Sciences and Sports, Macao Polytechnic Institute, Macao, China; pedrofong@ipm.edu.mo (P.F.); p1505700@ipm.edu.mo (C.-H.H.); p1505748@ipm.edu.mo (C.-C.I.); p1505792@ipm.edu.mo (P.-I.S.)

**Keywords:** antibacterial phytochemicals, antibiotic resistance, bacterial protein targets, *Helicobacter pylori* inhibition, molecular docking

## Abstract

*Helicobacter pylori* infection is a WHO class 1 carcinogenic factor of gastric adenocarcinoma. In the past decades, many studies have demonstrated the increasing trend of antibiotic resistance and pointed out the necessity of new effective treatment. This study was aimed at identifying phytochemicals that can inhibit *H. pylori* and possibly serve as adjuvant treatments. Here, in silico molecular docking and drug-like properties analyses were performed to identify potential inhibitors of urease, shikimate kinase and aspartate-semialdehyde dehydrogenase. These three enzymes are targets of the treatment of *H. pylori*. Susceptibility and synergistic testing were performed on the selected phytochemicals and the positive control antibiotic, amoxicillin. The in-silico study revealed that oroxindin, rosmarinic acid and verbascoside are inhibitors of urease, shikimate kinase and aspartate-semialdehyde dehydrogenase, respectively, in which, oroxindin has the highest potency against *H. pylori*, indicated by a minimum inhibitory concentration (MIC) value of 50 μg/mL. A combination of oroxindin and amoxicillin demonstrated additive effects against *H. pylori*, as indicated by a fractional inhibitory concentration (FIC) value of 0.75. This study identified phytochemicals that deserve further investigation for the development of adjuvant therapeutic agents to current antibiotics against *H. pylori*.

## 1. Introduction

*Helicobacter pylori* infections may cause chronic gastritis, peptic ulcer, gastric cancer and other non-gastric related disorders, such as lymphoma [1]. The World Health Organisation has considered *H. pylori* as a class 1 carcinogen [2]. A recent study reported that until 2015, around 4.4 billion people were infected with *H. pylori*, and the prevalence rate in Africa, Latin America and Asia is substantially increasing [3]. Thus, *H. pylori* is a global public health concern. The main treatment option for *H. pylori* is the standard triple therapy, combining two antibiotics with one proton pump inhibitor, such as clarithromycin and amoxicillin with omeprazole [4]. Due to the development of a drug-resistant strain, the failure rate of triple therapy has increased to more than 20% in many parts of the world [5]. This causes the use of higher doses or more drugs, such as the quadruple therapy, and this has led to a higher risk of side effects. To solve this problem, some researchers began to combine phytomedicines with triple therapy [6,7]. Some of their results showed the ability of phytomedicine to reduce side effects and decrease the treatment failure rate; however, their pharmacological mechanism of action is unclear.

Many pharmacological targets against an *H. pylori* infection have been identified, and they are generally related to *H. pylori*’s morphological structure, survival mechanisms and toxic factors. One well-known example of these targets is urease. *H. pylori* requires urease and the H^+^-gated urea channel to survive in the low pH environment of human gastric fluid. Ureases help to generate a layer of ammonia, which neutralises the stomach acid and resists the damage caused by acidic environments [8]. Another identified pharmacological target is shikimate kinase, which is necessary for the synthesis of aromatic amino acids of *H. pylori* as it catalyses the formation of shikimic acid in the shikimate pathway [9]. The third example is aspartate-semialdehyde dehydrogenase, which is an essential enzyme of *H. pylori* that produces some major amino acids and metabolites [10,11]. This study was aimed to identify inhibitors of these three targets.

Many phytomedicines had been investigated for their anti-*H. pylori* abilities through in vitro, in vivo and randomised control clinical studies [6]. Some of them have shown promising results [12,13,14]; however, their active components and pharmacological mechanisms remain unclear. One of the many examples would be the study of the Chinese patented medicine, Wenweishu [15]. This randomised, controlled, multicentre study involved 642 patients with *H. pylori* infections and peptic ulcers. The results demonstrated that the use of Wenweishu together with the standard triple therapy can significantly increase the healing rate, but the eradication rate was not statistically different. Another example is the in vitro study of the leaf extract of *Allium ascalonicum* [14]. which contains mixtures of alkaloids and cardiac glycosides that can inhibit urease activity, and hence produce anti-*H. pylori* effects. One of the downfalls of using plant extracts as medicine is the imprecise type and amount of the active ingredients. This is because many factors could affect the number of active ingredients of a plant, including climate, soil type and harvesting time [16]. Also, the mixture of ingredients in extracts may bind to multiple pharmacological targets, producing both desired and undesired biological responses. Hence, identifying the anti-*H. pylori* compounds in these plants may help to produce more predictable responses and accurate dosing regimens.

In silico molecular docking and drug-like properties analysis is an efficient method to screen bioactive compounds from a pool of phytochemicals [17]. Docking can simulate the interactions between a ligand and protein, calculate their binding energies and predict the possibility of whether a compound may bind to a pharmacological target, such as an enzyme. Drug-like properties analysis screens the phytochemicals with desired pharmacokinetic properties, including the absorption, distribution, metabolism, excretion and toxicity [18]. Docking has been widely used to identify bioactive compounds for further in vitro and in vivo studies. More importantly, docking has identified inhibitors for the three pharmacological targets in this study, urease [19], shikimate kinase [9,20], and aspartate-semialdehyde dehydrogenase [11]. Using in silico and in in vitro experiments, this study aimed to identify bioactive phytochemicals that can inhibit *H. pylori*.

## 2. Results and Discussion

This study performed molecular docking and drug-like properties analysis to identify phytochemicals that may inhibit the three pharmacological target enzymes of *H. pylori*: urease, shikimate kinase and aspartate-semialdehyde dehydrogenase. Phytochemicals were selected from the Traditional Chinese Medicine Systems Pharmacology Database and Analysis Platform (TCMSP). GOLD v5.5 was used as the docking suite. The identified inhibitors were oroxindin, rosmarinic acid and verbascoside, respectively. In vitro susceptibility and synergistic testing against *H. pylori* were then performed on these three phytochemicals and the parallel positive control antibiotic (amoxicillin) to calculate their minimum inhibitory concentration (MIC) and fractional inhibitory concentration (FIC) values.

### 2.1. In Silico Simulations

**Figure 1:**
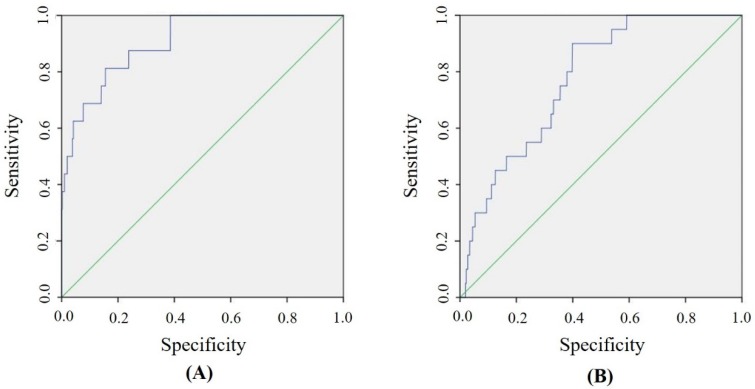
Receiver operating characteristic (ROC) curves of the docking results for the compounds from the Zinc In Man (ZIM) database were (**A**) urease with AUC = 0.90 and (**B**) shikimate kinase with AUC = 0.77. The diagonal green line indicates an area under curve (AUC) value of 0.50, meaning results occurred by chance. An AUC value between 0.7 and 1.0 indicates the results had reliable sensitivity and specificity.

The accuracy of the docking procedures varies substantially between different docking suites. Here, we validated our docking procedures on urease and shikimate kinase using receiver operating characteristic (ROC) analysis; their area under the curve (AUC) values were 0.90 and 0.77, respectively (Figure 1). An AUC value of 0.7 or above indicates a reliable docking procedure [21,22]. Hence, our docking approaches have reliable predictive power.

**Figure 2:**
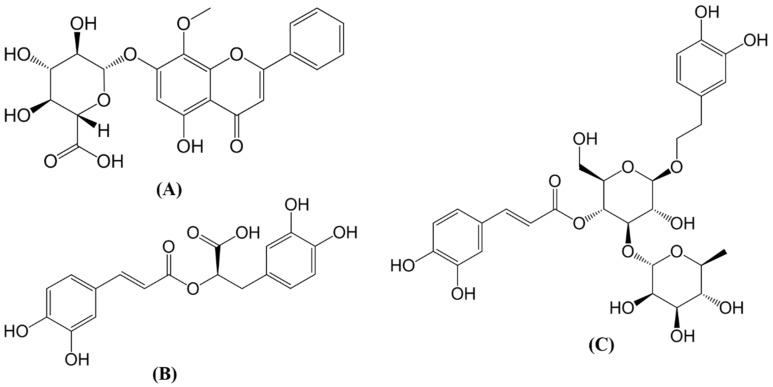
Chemical structures of (**A**) oroxindin, (**B**) verbascoside and (**C**) rosmarinic acid.

Molecular docking and drug-like properties analysis were performed on the three target enzymes to select phytochemicals for in vitro studies. The drug-like properties were classified into three categories: ADME (absorption, distribution, metabolism and excretion), physicochemical and drug safety (see Section 3.3). We selected one phytochemical with the finest balance between the predicted binding affinity and drug-like properties for each target enzyme. The three selected phytochemicals were oroxindin, verbascoside and rosmarinic acid (Figure 2).

Oroxindin obtained a high urease binding score of 84.9, which is comparable to most of the known urease inhibitors with binding scores ranging from 57.1 to 111.8. The botanical source of oroxindin is Radix Bupleuri, which has been shown to have an anti-*H. pylori* effect [23]. However, the active ingredients of Radix Bupleuri responsible for the anti-*H. pylori* effect have not been identified. Oroxindin could be one of Radix Bupleuri’s active phytochemicals against *H. pylori*. Oroxindin has similar chemical structures with similar functional groups to quercetin and baicalin, which have been experimentally demonstrated with *H. pylori* inhibitors [24,25,26]. Regarding drug-like properties, oroxindin was predicted to be a non-inhibitor to all Cytochrome P450 enzymes and hERG, as well as a non-central nervous system (CNS) penetrant (Table 1). This means oroxindin is unlikely to have drug–drug interactions, cardiotoxicity and CNS side effects. Oroxindin also has high water solubility and poor human intestinal absorption (HIA, Table 1). These results indicate that oroxindin can dissolve, spread and reach *H. pylori* in the human gastric region without too much systematic absorption into the bloodstream.

The docking score of rosmarinic acid on shikimate kinase was 79.1, which is comparable to the scores of many known inhibitors which range from 57.8 to 89.2. Rosmarinic acid achieved an excellent drug-like profile, with appropriate ADME, physicochemical and drug safety properties (Table 1). Similar to oroxindin, rosmarinic acid is highly water soluble, has poor HIA, and is unlikely to produce drug–drug interactions, cardiotoxicity or CNS side effects (Table 1). The botanical sources of rosmarinic acid are *Melissa officinalis* and *Ocimum basilicum*; *Melissa officinalis* exhibits a gastroprotective effect against gastric ulcers in animal studies [27], and *Ocimum basilicum* has been shown to significantly inhibit *H. pylori* in an in vitro study [28]. Again, the pharmacological mechanism of these two botanical sources is not clear and the active ingredients are unknown. Rosmarinic acid has demonstrated its antimicrobial effects on many bacteria strains, including *Enterobacter* species [29], *Escherichia coli* [30], *Aspergillus niger* [31], and many more [32]. Rosmarinic acid also has synergistic effects with amoxicillin, vancomycin and ofloxacin against *Staphylococcus aureus* [33]. Hence, rosmarinic acid has a higher possibility of inhibiting *H. pylori* than many other phytochemicals and was selected for further in vitro studies.

Verbascoside was the phytochemical selected to target aspartate-semialdehyde dehydrogenase. It obtained a docking score of 82.31 and ranked 11th out of 4450 herbal compounds. Similar to the other two phytochemicals, verbascoside has acceptable drug-like properties, such as good water solubility, non-CNS penetrant and non-inhibitor to all CYPs and hERG. (Table 1) High contents of verbascoside were shown in the leaf extracts of *Aloysia triphylla*, which exerted antibacterial effects against *Proteus mirabilis* [34], *Staphylococcus aureus* [35] and *H. pylori* [36] in in vitro studies. A recent study also pointed out the synergistic effects of verbascoside and gentamicin against *Staphylococcus aureus* and *Escherichia coli* [37]. Verbascoside demonstrated gastroprotective effects in an animal study by inhibiting the excretion of gastric acid through blocking H^+^/K^+^-ATPase. This indicates that verbascoside has the potential to mimic the action of a proton pump inhibitor in *H. pylori* treatment [38].

Due to the above-mentioned high docking scores, favourable drug-like properties and antibacterial effects suggested by the literature, we selected oroxindin, verbascoside and rosmarinic acid for further in vitro studies. According to our knowledge and literature searches in various databases, including the Cochrane Library, Embase, Medline and the China National Knowledge Infrastructure database, there is no experimental proof on the inhibitory effects of these three phytochemicals against *H. pylori*.

### 2.2. In Vitro Susceptibility and Synergistic Testing

All the three selected phytochemicals, oroxindin, verbascoside and rosmarinic acid had anti-*H. pylori* effects (Table 2). Among them, oroxindin had the highest potency, indicated by the lowest MIC value of 50 μg/mL. However, it is still less potent than the positive control antibiotic, amoxicillin, which obtained a MIC value of 0.250 μg/mL (Table 2). Regarding the synergistic testing, oroxindin demonstrated additive effect with each of the other two phytochemicals and amoxicillin, indicated by the value of 0.75 (Table 3). There were no synergistic or antagonistic effects observed in all the other combinations.

The MIC value of oroxindin was 50 μg/mL, which is comparable with some well-known anti-*H. pylori* phytochemicals, such as scopolin (50–100 μg/mL) [39], chelerythrine (25–100 µg/mL) [40], and protopine (25–100 µg/mL) [40]. However, the MIC value of oroxindin is larger than some high potency phytochemicals, such as berberine (0.78–25 μg/mL) [40], fuscaxanthone (16.3–131.2 μg/mL) [41], palmatine (3.12–6.25 μg/mL) [42] and allicin (6 μg/mL) [43]. Certainly, MIC values can be affected by many factors, such as experimental procedures and reagents; thus, it is not reasonable to directly compare MIC values obtained from different studies. However, comparing these values provides a rough overview of their potency, signifying the anti-*H. pylori* effect of oroxindin.

Although many phytochemicals have suggested anti-*H. pylori* effects, many of them have undesirable side effects or unfavourable drug-like properties [44]. For example, allicin has superior MIC values (6 μg/mL) [43], but it can cause gastric side effects, such as heartburn, diarrhoea and nausea. It also has antithrombotic properties and can prolong bleeding time [45]. Another example is palmatine, which can significantly inhibit *H. pylori* and has antiviral, anticancer and antihyperlipidemic effects [42]. However, it has noticeable DNA toxicity and complex interactions with liver metabolic enzymes [46]. In contrast, our validated in silico study suggested that oroxindin has poor human intestinal absorption, indicating the amount of oroxindin that can be absorbed into the bloodstream is low. Hence, the risk of causing systematic side effects and the risk of interacting with metabolic enzymes in other organs is low. Also, an *H. pylori* infection can irritate the stomach and cause gastritis, and oroxindin has demonstrated its gastroprotective effects by suppressing the inflammatory response and conserving the gastric barrier function [47].

The MIC values of oroxindin, verbascoside and rosmarinic acid are higher than the positive control, amoxicillin (Table 2). This indicates the phytochemicals are less potent than one of the current antibiotic drugs of *H. pylori*. The current recommended treatment of *H. pylori* is triple therapy, which contains two antibiotics, such as amoxicillin, clarithromycin, levofloxacin and metronidazole. In a study by Lee et al. [48], in vitro susceptibility tests were performed on the same strains of *H. pylori* (ATCC 43504) as in this study, and the MIC values of amoxicillin, clarithromycin and metronidazole were 0.029, 0.06 and 21.6 μg/mL, respectively. These referenced MIC values suggested that oroxindin (MIC: 50 μg/mL) may have less potency than amoxicillin and clarithromycin, but higher potency than metronidazole. For some antibiotic resistant clinical strains, these MIC values were much higher. Recent studies from different countries have reported that MIC values of amoxicillin, clarithromycin and metronidazole on clinical *H. pylori* strains were 256 µg/mL [49,50]. The resistance mechanisms of these antibiotics do not seem to be related to the three target enzymes involved in this study, urease, shikimate kinase and aspartate-semialdehyde dehydrogenase [51]. Hence, theoretically, oroxindin, verbascoside and rosmarinic acid should have similar potencies in both resistant and non-resistant strains. The MIC value of oroxindin (50 µg/mL) could be better than that of antibiotics in the resistant strains, and produce comparable or enhanced anti-*H. pylori* effects. Also, as the anti-*H. pylori* pharmacological mechanisms are different between the antibiotics and phytochemicals, the co-administration of them may improve their efficiency and reduce side effects by decreasing the dosage typically administered.

This study demonstrated an additive inhibitory effect between oroxindin and amoxicillin. This could be due to their different pharmacological mechanisms, in which amoxicillin is a beta-lactam antibiotic that binds to penicillin-binding proteins and inhibits the synthesis of peptidoglycan of *H. pylori* cell walls. Whereas, this in silico study suggests that oroxindin binds to *H. pylori* urease, which is responsible for converting stomach urea to ammonia and neutralise the gastric acid and protect *H. pylori* from the surrounding strong acids [52]. The suggested pharmacological mechanisms of verbascoside and rosmarinic were also different from that of amoxicillin, however, there were no synergistic or additive effects. We believe this is due to their large differences between their potency, indicated by their MIC values (Table 2). The very potent amoxicillin would have killed most *H. pylori* before the phytochemicals produced a significant effect. However, in an antibiotic resistant strain, the difference between their potency will be reduced, and hence there will be a higher chance for producing synergistic or additive effects. Certainly, further studies on clinically obtained antibiotic resistance *H. pylori* strains are required to prove this theoretical idea.

## 3. Materials and Methods

This study performed molecular docking and drug-like properties analysis to select phytochemicals with desired pharmacokinetic properties that may interact with three anti-*H. pylori* targets, urease, shikimate kinase and aspartate-semialdehyde dehydrogenase. In vitro anti-*H. pylori* assays were performed to test the inhibitory abilities of these selected phytochemicals. We also investigated the synergistic anti-*H. pylori* effects between the phytochemicals and an antibiotic.

### 3.1. Molecular Docking

Docking has been used to identify inhibitors from phytochemicals by analysing the interactions between the target protein and ligands. Several molecular docking software programmes have been successfully applied in computational drug design (CADD). Here, the automated docking suite, GOLD v5.5 [53,54] was used to study the binding potential between the three target enzymes, urease, shikimate kinase and aspartate-semialdehyde dehydrogenase, with the selected pool of phytochemicals. According to the Traditional Chinese Medicine Systems Pharmacology Database and Analysis Platform (TCMSP) [55], 38 herbs can inhibit urease, and these herbs contain 5015 phytochemicals. All these phytochemicals were docked to the urease X-ray crystallographic structure with PDB code 1E9Y [56]. All ligands, ions and water were removed before the docking simulations. The ChemPLP scoring function [57], genetic algorithms with 100% search efficiency, no early termination, the slow option with high accuracy and the default parameters were used for all the docking simulations. Atoms within an area of 6 Å of the cognate ligands in the X-ray crystallographic structures were set as the binding sites. For shikimate kinase, 14 herbs were identified as inhibitors, and these herbs contained 1548 phytochemicals. The structure of shikimate kinase employed was PDB: 3N2E [9]. As there was no *H. pylori* X-ray crystallographic structures of aspartate-semialdehyde dehydrogenase, the homology structure built by SWISS-MODEL [58] with sequence identity and sequence similarity of 48.33% and 0.41, respectively, was employed for docking. A report with details of the sequence alignment can be obtained from https://swissmodel.expasy.org/repository/uniprot/O25801#none. The number of herbs and phytochemicals involved in the aspartate-semialdehyde dehydrogenase docking simulations were 32 and 4541, respectively. The docking procedures of shikimate kinase and aspartate-semialdehyde dehydrogenase were the same as that of the urease.

### 3.2. Validation of Molecular Docking Methods

The accuracy of molecular docking varies substantially between different docking algorithms, scoring functions and the type of protein–ligand interactions [59]. The docking software that we used in this study was GOLD v5.5 [53,54], which has been extensively tested across various proteins and ligands by both the software supplier (The Cambridge Crystallographic Data Centre, https://www.ccdc.cam.ac.uk) and researchers [17]. GOLD has also been successfully used to perform docking experiments on the targeted enzymes in this study, urease, shikimate kinase and aspartate-semialdehyde dehydrogenase [9,11,19,20]. However, we still believe it is necessary to further evaluate its specific accuracy on the two targeted enzymes using receiver operating characteristic (ROC) analysis. Here, both the X-ray crystallography structures of urease (PDB: 1E9Y) and shikimate kinase (PDB: 3N2E) were docked separately to the 11,421 ligands of the Zinc In Man (ZIM) database [60]. A total of 24 experimentally approved urease inhibitors and 20 shikimate kinase inhibitors with IC_50_ less than 100 µM were also docked with the urease and shikimate kinase, respectively. These inhibitors served as the ‘positive’ hits, whereas the 11,421 ZIM ligands were ‘negative’ hits. ROC analysis statistically revealed the ability of the docking methods to distinguish between the ‘positive’ and ‘negative’ ligands. All the experimentally approved inhibitors were identified from the BindingDB database [61], and our literature searches on the Cochrane Library, Embase, Medline and the China National Knowledge Infrastructure database. For aspartate-semialdehyde dehydrogenase, only two experimentally approved antibacterial inhibitors were found in the literature and databases [10], and this small number of inhibitors did not provide sufficient statistical power for the ROC analysis to validate the docking procedures.

### 3.3. Drug-Like Properties Analysis

Drug-like properties analyses of the phytochemicals were performed using the ACD/Percepta 14.0 software [62]. The analysis involved three categories of properties: ADME, physicochemical and drug safety. ADME predicts factors that affect absorption, distribution, metabolism and excretion. These factors include human intestinal absorption (HIA), passive permeability across Caco-2 cell monolayers and plasma protein binding [63]. Physicochemical properties, such as molecular weight, the number of H-bond donors/receptors, solubility, log P and predefined lead-like categories were predicted to evaluate the pharmacokinetic and pharmacodynamic properties of the phytochemicals. Drug safety properties evaluated the toxicity of the phytochemicals, including the probability of causing mutagenicity (positive Ames), cardiotoxicity (human ether-à-go-go-related gene, hERG) and drug–drug interactions (Cytochrome P450 regioselectivity) [64,65]. The performance of all these predictions were successfully analysed by the supplier company (ACDS/Labs), which is available at https://www.acdlabs.com/products/percepta/index.php.

### 3.4. Susceptibility Testing

The minimum inhibitory concentration (MIC) was used to evaluate the inhibitory abilities of the selected phytochemicals against *H. pylori*. MIC is the lowest concentration of the phytochemicals required to inhibit the visible growth of *H. pylori*. The strain of *H. pylori* used in this study was ATCC-43504, which were recovered according to the supplier product information sheet (https://www.atcc.org/~/ps/43504.ashx), wherein the frozen strain was thawed in a water bath at 37 ℃ and inoculated in solutions (100 μL) containing 6% sheep blood Colombian agar (Huankai Microbial, Guangdong, China) and 5% foetal bovine serum (Huankai Microbial, Guangdong, China) in a facultative anaerobic environment for 3 days. The *H. pylori* solution were added to cryopreservation media containing brain heart infusion broth and glycerine and stored at −80 ℃. To identify the *H. pylori* strain, a combination of the test was performed. The microscopy visualisation of colony morphology was used to assess the needle-like translucent appearance of *H. pylori*. Three biochemical assays, urease, oxidase and catalase tests (Huankai Microbial, Guangdong, China), were performed to confirm which *H. pylori* strains were present.

After identification, colonies of *H. pylori* were added to 0.85% normal saline to produce a solution with 0.5 (approximately 1.5 × 10^8^ CFU/mL) turbidity under the microbial turbidimeter (DensiCHEK Plus, bioMerieux, USA). The solution was then diluted 50 times to make an approximately 3.0 × 10^6^ CFU/mL *H. pylori* turbidity solution.

The inhibitory effects of the selected phytochemicals were obtained using microdilution methods, in which sterile Brucella broth (HopeBio, Qingdao, China) was used to dilute each of the most concentrated phytochemical solutions by six half-dilutions to produce solutions with a range of different concentrations. Two microliters of the prepared *H. pylori* McFarland Turbidity solution (approximately 3.0 × 10^6^ CFU/mL) was then added to each of the phytochemical solutions and incubated at 37 ℃.

The MIC experiments were performed in sterile 96-well microliter plates (ChunBo Biologics, Haimen, China) at 37 ℃. The optical density (OD) values were collected from a spectrophotometer (Multiskan™ GO, Thermo Fisher Scientific) at an absorbance of 540 nm after 72 h. These experiments were performed with quality, negative and blank controls, which contained no *H. pylori* or phytochemicals, only *H. pylori* (no phytochemicals) and only phytochemicals (no *H. pylori*), respectively. These experiments were also performed with a commonly prescribed antibiotic (amoxicillin) for *H. pylori* eradication. Amoxicillin served as a parallel positive control, and the MIC value of amoxicillin was calculated for comparison with those of the phytochemicals. All experiments followed the guidelines from the Clinical and Laboratory Standards Institute [66].

The MIC_90_ value is the minimum concentration of phytochemicals that inhibit 90% of *H. pylori*. The inhibition percentage was calculated using the following equation:

$$\mathrm{Inhibition}\;\mathrm{percentage}\;\left(\%\right)\;=\;\left(\frac{\mathrm{OD}\;\mathrm{value}\;\mathrm{of}\;\mathrm{the}\;\mathrm{sample}-\mathrm{OD}\;\mathrm{value}\;\mathrm{of}\;\mathrm{blank}\;\mathrm{control}\;}{\mathrm{OD}\;\mathrm{value}\;\mathrm{of}\;\mathrm{the}\;\mathrm{negative}\;\mathrm{control}-\mathrm{OD}\;\mathrm{value}\;\mathrm{of}\;\mathrm{quality}\;\mathrm{control}}\right)\;\times \;100\%$$

### 3.5. Synergistic Testing

Synergistic testing was used to study whether the additive effect of two compounds or antibiotics was superior to that of the effect of the individual compounds. An additive effect may help to reduce the dose of each compound and may reduce adverse pharmacological effects. Here, the MIC values of the phytochemicals and antibiotics obtained from the susceptibility testing were used to calculate the concentrations required for the synergistic testing. Various combinations of two compounds or antibiotics at different concentrations were tested. Their concentrations were 1/8 MIC, 1/4 MIC, 1/2 MIC, 1 MIC and 2 MIC. The checkerboard methods [67] were used to calculate the fractional inhibitory concentration (FIC) values between the phytochemicals and amoxicillin against *H. pylori*. FIC values are measurements of synergistic, additive or antagonistic effects. An FIC value of less than 0.5 indicated synergistic action, between 0.5 and 1.0 indicated additive action, 1.1 to 4.0 meant indifferent and larger than 4.0 indicated antagonism [68,69]. All experiments used to calculate FIC were performed in the same manner as that of the MIC. The FIC values were calculated using the following equation:

$$\mathrm{FIC}\;\mathrm{value}\;=\;\left(\frac{MIC\;of\;compound\;A\;in\;combination}{\;MIC\;of\;compound\;A\;alone}\;+\;\frac{MIC\;of\;compound\;B\;in\;combination}{\;MIC\;of\;compound\;B\;alone\;}\right)$$

### 3.6. Statistical Analysis

A paired t-test was used to calculate the *p*-values for comparing the OD, MIC and FIC values of the sample groups and control groups at different concentrations. A *p*-value of less than 0.05 was considered to be statistically significant. The SPSS software (version 22.0, IBM Corp., Armonk, NY) was used.

## 4. Conclusions

This study performed validated in silico techniques to identify a urease, a shikimate kinase and an aspartate-semialdehyde dehydrogenase inhibitor from 5015, 1548 and 4541 phytochemicals, respectively. The three identified inhibitors with appropriate drug-like properties were oroxindin, verbascoside and rosmarinic acid. In our in vitro susceptibility testing, all three phytochemicals were shown to have anti-*H. pylori* effects, in which oroxindin had the highest potency. Their MIC values were higher than the current anti-*H. pylori* treatment, amoxicillin. Hence, their potential use as monotherapy of *H. pylori* treatment cannot be justified here. In our synergistic testing, oroxindin demonstrated its additive effects with amoxicillin. We believe further investigations on these phytochemicals as an adjuvant therapy with current antibiotics treatment on both resistant and non-resistant strains are well-intentioned.

## Figures and Tables

**Table 1:** Docking scores and drug-like properties of the phytochemicals.

	Oroxindin	Verbascoside	Rosmarinic acid
Docking Score ^1^	84.9	79.1	82.3
MW	460.4	624.6	360.3
log P	-0.03	0.75	1.60
Aqueous solubility (mg/mL)	1000	15.7	1000
Caco-2	0.0 × 10^−6^	0.1 × 10^−6^	0.2 × 10^−6^
PPB (%)	89	53	74
CNS (cm/s)	−6.49	−5.22	−4.96
HIA (%)	1	9	8
Ames	0.49	0.44	0.34
hERG	0.33	0.28	0.21
CYP1A2	NI	NI	NI
CYP2C9	NI	NI	NI
CYP2C19	NI	NI	NI
CYP2D6	NI	NI	NI
CYP4A4	NI	NI	NI

^1^ The docking scores of oroxindin, verbascoside and rosmarinic acid corresponded to urease, aspartate-semialdehyde dehydrogenase and shikimate kinase, respectively. MW: molecular weight; log P: octanol–water partition coefficient at 25 ℃ under standard conditions (optimal value: −1.00 to 4.20); aqueous solubility was calculated at pH 6.4 (>0.1 indicates soluble); Caco-2 predicts passive intestinal permeability (≤1.00 indicates poorly permeable); PPB represents plasma protein binding; central nervous system (CNS) values of ≤ −3.50 indicates non-central nervous system penetrant; HIA is human intestinal absorption (≤30% indicates poorly absorbed); Ames estimates mutagenic potential (≤0.33 indicates non-mutagenic, 0.33–0.67 is undefined, >0.67 is mutagenic); hERG values of less than 0.33 indicates non-inhibitor of hERG channel and has low risk of cardiotoxicity; CYP is Cytochrome P450 and NI means non-inhibitor.

**Table 2:** Minimum inhibitory concentration (MIC) values and inhibition percentage of test samples for ATCC-43504.

Test Samples	MIC90 (μg/mL)	Inhibitory %
Oroxindin	50	97.6 ± 3.5
Verbascoside	1200	97.7 ± 3.2
Rosmarinic acid	800	96.9 ± 6.4
Positive control 1	0.250	92.0 ± 2.2

^1^ The parallel positive control was amoxicillin.

**Table 3:** Fractional inhibitory concentration (FIC) values of test samples for ATCC-43504.

Test samples	FIC values	Outcome
Oroxindin plus amoxicillin	0.750	additive effect
Oroxindin plus verbascoside	0.750	additive effect
Oroxindin plus rosmarinic acid	0.750	additive effect
Verbascoside plus amoxicillin	1.125	indifference
Rosmarinic acid plus amoxicillin	1.125	indifference
Verbascoside plus rosmarinic acid	1. 250	indifference
